# 

**Published:** 1899-07-29

**Authors:** 


### NOTICE TO CORRESPONDENTS.

All MS., Letters, Books for Review, and other matters intended for the Editor should be addressed The Editor, "The Hospital," The Hospital Building, 28 & 29, Southampton Street, Strand, W.C.
The Editor cannot undertake to return rejected MS., even when accompanied by stamped directed envelope.

**NOTICE.—Vol. XXV. of "The Hospital" (October, 1898, to March, 1899), in handsome cloth binding, is now on sale at the Office of this Journal, price 7s. 6d. Cases for binding the Numbers contained in this Volume 1s. 6d. Index to Vol. XXV., 2½d., post free.**
**The Monthly Part for August will be ready on August 1st. Price 6d., by post 8d.**

### Scraps and Gleanings.

A sum of £5,000 is required to enable the Bedford County Hospital to start free from debt.

In our issue of April 15th last, in our note in this column as to the Sydney Hospital, New South Wales, it should have been stated that 3,904 indoor cases were treated in 1898.

A Military Tournament on behalf of the Cardiff Infirmary is shortly to be held, and a committee has been formed to draw up a programme. A tournament on behalf of the Cardiff Seamen's Hospital is also to be held on August 23rd.

The recommendation to add a new isolation ward to Milton Hospital has been agreed to by the Portsmouth Town Council, and at the same meeting Dr. McGregor was appointed medical superintendent at a salary of £150 per annum.

A new Convalescent Home at Castle Donington, established by the minister and church of Melbourne Hall, near Leicester, has just been opened. It is intended for the poorer women and girls who need a change of scene and air but cannot afford to obtain it, and it is unsectarian.

